# Low dose sacubitril/valsartan is effective and safe in hemodialysis patient with decompensated heart failure and hypotension: A case report

**DOI:** 10.1097/MD.0000000000029186

**Published:** 2022-04-15

**Authors:** Yunlin Feng, Wenhua Li, Hongjun Liu, Xiuling Chen

**Affiliations:** a Nephrology Department, Sichuan Provincial People's Hospital, Chengdu, China; b Medical School of University of Electronic Science and Technology of China, Chengdu, China; c Ultrasound in Cardiac Electrophysiology and Biomechanics Key Laboratory of Sichuan Province, Sichuan Provincial People's Hospital, Chengdu, China; d Nephrology Department, Anyue County People's Hospital, Ziyang, China.

**Keywords:** angiotensin-receptor neprilysin inhibitor, case report, heart failure, hemodialysis, hypotension

## Abstract

**Rationale:**

Severe heart failure in chronic hemodialysis (HD) patients is a great treatment challenge. Here we reported a chronic HD patient with the lowest ejection fraction reported so far and hypotension who well tolerated and benefited from angiotensin-receptor neprilysin inhibitor (ARNI) treatment.

**Patient concerns:**

This case was a 67 year old lady with decompensated heart failure and hypotension who was on regular HD. Intensified hemofiltration failed to improve her heart failure symptoms and was also retarded by hypotension.

**Diagnosis:**

Chronic HD with decompensated heart failure.

**Interventions:**

In addition to regular HD, low does sacubitril/valsartan was initiated and titrated from 12/13 mg to 24/26 mg twice daily.

**Outcomes:**

Sacubitril/valsartan treatment was well tolerated and did not affect ultrafiltration during HD treatment. Transthoracic echocardiology at 3 months after initiation of ARNI treatment indicated significant improvement of both systolic and diastolic cardiac function. The patient has improved from New York Heart Association class 4 to class 2.

**Lessons:**

Low does ARNI treatment could effectively improve cardiac function in HD patients with heart failure and hypotension. It was also safe and well tolerated.

## 1 Introduction

Severe heart failure in chronic hemodialysis (HD) patients is a great challenge to treating physicians.^[1]^ The removal of excess fluid to relive cardiac congestion is retarded by rapid drop of blood pressure, which is usually intolerable in the presence of hypotension. Increasing the duration and frequency of individual dialysis session^[2]^ and continuous renal replacement therapy^[1]^ are validated options in such circumstances, however, these regimens require strong logistic support and are often unrealistic in ordinary clinic HD centers.

Sacubitril/valsartan is an angiotensin-receptor neprilysin inhibitor (ARNI)^[3]^ that has been approved by the National Medical Products Administration for treatment of heart failure with reduced ejection fraction (HFrEF) in 2017 and essential hypertension in 2021.^[4]^ However, the IFU does not recommend ARNI treatment for patients with estimated glomerular filtration rate less than 30 mL/min/1.73 m^2^. Its efficacy and safety in end stage renal disease population needs more evidence. In addition, the antihypertensive effect of ARNI theoretically limits its use in hypotensive HD patients who rely on fluid removal during 4 hours of treatment to maintain water hemostasis.

Here we reported a chronic HD patient with the lowest ejection fraction reported so far and hypotension who well tolerated and benefited from ARNI treatment, aiming to provide more evidence for the efficacy and safety of ARNI in this population.

## 2 Case presentation

The patient was a 67-year-old lady who had been on chronic HD for 2 years. Her primary renal disease was chronic glomerulonephritis. The patient also had hypertension for 12 years before initiation of HD and her blood pressure had been stable around 140/90 mm Hg at 30 mg nifedipine controlled-release tablet and 80 mg valsartan capsule once daily. Family history and social-psycho history were unremarkable. One month before the admission, the patient gradually developed shortness of breath, palpitation and orthopnea during nights. Her dialysis frequency was changed to 4 time a week and the symptoms were only modestly improved. The blood pressure dropped rapidly after intensive HD treatment. Five days before admission, the patient passed out once at her local HD center. The blood pressure at that time was 90/50 mm Hg. The tremor of her arterial-venous fistula vanished after that incident, and subsequent vascular doppler ultrasound examination confirmed thrombosis within cephalic vein at 3 cm proximal to fistula anastomosis. Percutaneous catheterization of the internal jugular vein was conducted to provide HD access and her antihypertensive treatment was stopped. The patients was then transferred to our hospital for further treatment.

Vital signs at admission were temperature 36.6°C, blood pressure (BP) 105/80 mm Hg, heart rate 90 bpm, and respiratory rate 26 bpm. Pulse oxygen saturation at room air was 94% to 96%. Physical examination indicated tachypnea without jugular vein engorgement. There were not any lower extremity edema. Her medications at admission included oral α-keto acid (KETOSTERIL) 2520 mg 3 times daily and cinacalcet 25 mg once daily, and recombinant human erythropoietin 4000 unit 3 time weekly. Laboratory investigation after admission indicated plasma brain natriuretic peptide of 18,668.4 pg/mL. Transthoracic echocardiology indicated severe impairment of cardiac function with EF 18%, as well as moderate pulmonary hypertension, diastolic dysfunction, moderate mitral regurgitation, and moderate tricuspid regurgitation. The patient was rated as New York Heart Association class 4 heart failure.

We increased the HD frequency to 4 times per week. The ultrafiltration volume at each session was between 1500 and 2000 mL. Her symptoms could temporarily improve after HD treatment but worsened quickly on the next day. The plasma brain natriuretic peptide level reduced to 12,833.6 pg/mL at 5 days after admission. However, the efficacy of increasing ultrafiltration to improve her symptoms gradually reduced due to rapid drop of BP during HD session and frequent occurrence of hypovolemic symptoms including excessive sweating and lower limb muscle cramps. The lowest BP recorded during HD sessions was 86/45 mm Hg. The patient was not considered a candidate for mitraclip due to financial reasons. She then started sacubitril/valsartan (Entresto, Norvatis, Switzerland) treatment at a starting dose of 12/13 mg twice daily at 7 days after admission. The patient tolerated sacubitril/valsartan very well. Her predialysis BP kept stable at 100/70 mm Hg, and her symptoms significantly improved. We titrated her dose to 24/26 mg twice daily 3 days later. The HD frequency was reduced to 3 times a week. At 14 days after admission, the patients was discharged to her local HD center.

She continued with the regimen of sacubitril/valsartan 24/26 mg twice daily and regular HD session 3 times a week. Her predialysis BP kept stable at round 100/60 mm Hg. Her symptoms continued to improve, and she was able to quietly sleep in supine position from 3 weeks after ARNI treatment. She also had her arterial-venous fistula repaired afterwards. Her cardiac function improved from New York Heart Association class 4 to class 2 at the visit 3 months after starting ARNI treatment, and there were not episodes of worsening heart failure during the follow-up period. Transthoracic echocardiology indicated significant improvement of cardiac function parameters (see Table 1).^[5]^ The patient was very satisfied with her regimen.

**Table 1:** Transthoracic echocardiology results.

	Start of treatment	3 mo after starting	Normal range
LA (mm)	50	30	23–39
LV (D) (mm)	54	47	39–55
MV_E (m/s)	1.05	0.48	0.47–1.25
MV_A (m/s)	0.69	1.14	0.34–1.16
E/A ratio	1.52	0.42	0.44–2.00
MV_e (m/s)	0.07	N/A	0.03–0.15
MV_a (m/s)	0.05	N/A	0.06–0.14
E/e	15	N/A	0.04–0.15
EF (Simpson) (%)	18	60	52–78
RA (mm)	50	29	25–40
RV (mm)	26	21	15–28
MPA (mm)	28	21	14–26
PV (m/s)	0.46	0.72	0.59–1.33
PR (m/s)	2.41	N/A	<2.0
TR (m/s)	3.56	N/A	<2.8

E/A ratio = ratio of E wave to A wave, E/e = E/e ratio, EF (Simpson) = ejection fraction, LA = left atrium, LV (D) = left ventricle (diameter), MV_E = early wave of mitral flow, MV_A = atrial wave of mitral wave, MV_e = e wave velocity of basal lateral mitral annulus by TDI, MV_a = a wave velocity of basal lateral mitral annulus by TDI, MPA = main pulmonary artery (internal diameter), N/A = not available, PV = velocity of pulmonary artery, PVG = velocity gradient of pulmonary artery, PR = velocity of pulmonary regurgitation, PRG= velocity gradient of pulmonary regurgitation, RA = right atrium, RV = right ventricle, TR = velocity of tricuspid regurgitation, TRG = velocity gradient of tricuspid regurgitation.

## 3 Discussion

Here we reported a chronic HD patient with severe heart failure and hypotension who well tolerated and benefited from ARNI treatment. To our knowledge, the patient had the lowest ejection fraction reported so far in HD patients who received ARNI treatment for heart failure. This case further supported the efficacy and safety of ARNI in HD patients with HFrEF and hypotension.

End-stage heart failure is common in chronic HD population, especially in the elderly. Regular HD treatment is often retarded by rapid drop in blood pressure, thus worsening fluid accumulation which contributes to intolerable heart failure symptoms. The introduction of ARNI treatment has brought light on management of these patients. However, evidence for ARNI treatment in HD population is less abundant than that in essential hypertension population. Heyse et al^[6]^ firstly reported a similar case in 2019. In 2021, Lihua et al^[7]^ reported a group of 110 HD patients with HFrEF who had received ARNI treatment and concluded ARNI regimen was efficacy and safe in this population. This case had lower EF value than Heyse case (18% vs 35%) and lower blood pressure compared with the average level in Wang population. Low dose ARNI in this case was well tolerated, and had no negative effect on fluid removal during HD session. The patient returned to regular HD treatment, that is, 3 time per week, 4 hours per session, and her systolic blood pressure kept stable at around 100/60 mm Hg. The good tolerance and excellent improvement of symptoms further support ARNI protective effect for HD patients with heart failure and hypotension. Titrating from low doses of ARNI is an effective way to reach a balance between relieving heart congestion symptoms and maintaining a relative stable blood pressure to ensure uneventful HD treatment.

In patients with symptomatic heart failure, regular echocardiogram is recommended to monitor both structural and functional variables, including regional wall motion, ventricular function and size and EF, etc.^[8]^ Impaired EF is a well-known indicator for poor prognosis in heart failure patients.^[9]^ In this case, transesophageal echocardiography indicated both systolic and diastolic functions significantly improved after 3 months of treatment, consistent with the symptomatic improvement observed clinically. The patient herself was very satisfied with the treatment.

In conclusion, low does ARNI treatment could effectively improve cardiac function in HD patients with heart failure and hypotension. It was also safe and well tolerated.

## Acknowledgments

We sincerely thank the patient and her families for their trust. We also thank every health professional involved in caring this patient.

## Author contributions

FYL collected data and drafted the manuscript. LWH collected image data and drafted the manuscript. LHJ helped with data collection. CXL collected data and drafted the manuscript. All authors made critical revision to incorporate important intellectual content and approved the final manuscript.

**Conceptualization:** Yunlin Feng, Xiuling Chen.

**Data curation:** Yunlin Feng, Wenhua Li, Hongjun Liu, Xiuling Chen.

**Formal analysis:** Yunlin Feng, Wenhua Li, Xiuling Chen.

**Supervision:** Xiuling Chen.

**Writing – original draft:** Yunlin Feng, Wenhua Li, Hongjun Liu.

**Writing – review & editing:** Yunlin Feng, Wenhua Li, Hongjun Liu, Xiuling Chen.
